# Pneumomediastinum, pneumopericardium and subcutaneous emphysema following acute lymphoblastic leukemia and chemotherapy: A case report

**DOI:** 10.22088/cjim.12.0.379

**Published:** 2021

**Authors:** Mohammad Kajiyazdi, Amir Hossein Norooznezhad

**Affiliations:** 1 Pediatric Hematology and Oncology Ward, Bahrami Children Hospital, Tehran University of Medical Sciences, Tehran, Iran; 2 Medical Biology Research Center, Health Technology Institute, Kermanshah University of Medical Sciences, Kermanshah, Iran

**Keywords:** Pneumomediastinum, Pneumopericardium, Subcutaneous emphysema, Acute lymphoblastic leukemia

## Abstract

**Background:**

Pneumomediastinum and subcutaneous emphysema are mostly detected in non-malignant conditions such as certain infections, thoracic surgeries, and trauma. Although this condition is asymptomatic in most cases, sometimes it could be symptomatic and may even be lethal in some patients.

**Case Presentation:**

This letter reports a 9-year-old girl with acute lymphoblastic leukemia (ALL) on chemotherapy who developed pneumothorax with the clinical feature of respiratory distress for that a chest tube was inserted immediately. Following the insertion, pneumomediastinum and pneumopericardium developed in the patient. As the next step, a pericardium window was inserted by an expert heart surgeon. During these procedures, all the evaluations for any bacterial or fungal infection were negative. Unfortunately, the patient expired before any further complementary evaluations and it was not clear that the mentioned situation was a result of chemotherapy or ALL.

**Conclusion:**

Although pneumomediastinum and subcutaneous emphysema are rare in patients with ALL, authors strongly suggest clinicians consider them in any similar patients presenting respiratory signs/symptoms for faster onset of action.

Acute lymphoblastic leukemia (ALL) is the most common malignancy of childhood with the highest mortality rate among all cancers in patients aging less than 20 years old in the United States (1). Pneumomediastinum is defined by the presence of air in the mediastinum with the different possible origins such as may esophagus, lungs, or bronchi. Although in most cases, this phenomenon is following a trauma, it may also happen spontaneously with no history of trauma, medical procedures or any related surgery (2). The most important and common clinical sign of pneumomediastinum is chest pain which chest x-ray or thoracic computed tomography (CT) scan could use for diagnosis of suspicious cases. However, other signs such as dyspnea, coughing, cervical pain, and dysphagia are likely to happen as well. Moreover, in up to 70% of cases with the spontaneous pneumomediastinum, it may be accompanied by subcutaneous emphysema (3). It seems that the spontaneous pneumomediastinum is a self-limited phenomenon and could be caused by the alveolar rupture which is also known as the Macklin phenomenon. This phenomenon which shows a linear collections of air to the bronchovascular sheaths is caused by alveolar rapture (4). 

There are few case reports of pneumomediastinum in patients with leukemia. Although, there is still no exact etiology presented for this pathologic condition in ALL patients (4-7). This report aimed to add more clinical data to make a better insight for similar cases which could guide the clinicians.

## Case Presentation

**Fig 1:**
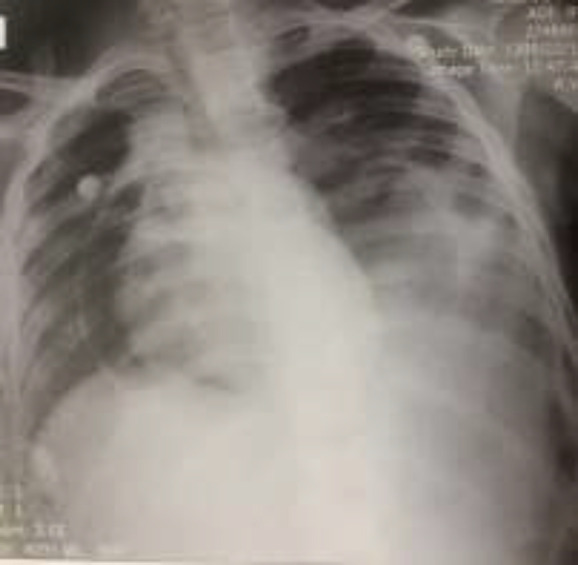
Chest X-ray showing pneumopericardium

The patient was a 9-year-old female (hospitalized in Bahrami Hospital, Tehran, Iran) diagnosed with pre-B cell lymphoblastic leukemia (BCR-ABL negative) who was on vincristine (weekly), daunorubicin (weekly), L-asparaginase (every other day), dexamethasone (3 times a day). At the end of the induction phase, the patient developed a sudden onset of chest pain, dyspnea, and tachypnea (respiratory rate: 55/min and O2 saturation of 80%) without fever (oral temperature: 37°C). Her respiratory distress worsened gradually. On physical examination, unilateral diminished breathing sounds on auscultation were recorded. Thus, a chest x-ray (posteroanterior view) was requested that revealed pneumothorax. Following surgical consultation, a chest tube was inserted for the patient by an expert surgeon. After the chest tube insertion, patient’s general condition improved and the oxygen saturation raised to more than 90% (respiratory rate: 60/min). However, she developed respiratory distress after a few hours. In the second chest x-ray, evidence such as mediastinal widening representing pneumopericardium was found (figure 1).

Due to the high-risk medical condition, the patient was transferred to the intensive care unit (ICU). Her general condition worsened and she developed hypotension (blood pressure: 70/55 mmHg). Upon a rising suspicion of bacteremia and sepsis in this situation, urine and sputum cultures were ordered for the patient which results came back negative for any bacterial growth. Although the cultures did not point to any clue of infection, intravenous meropenem (40mg/kg/day q8h; not to exceed 2gr), vancomycin (40mg/kg/day), and liposome amphotericin B (1mg/kg/day) were started for the patient. 

**Figure 2:**
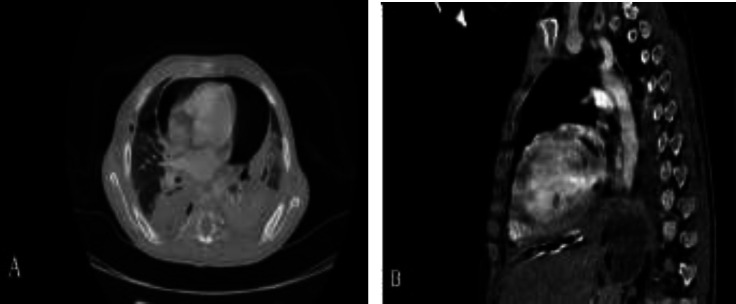
Contrast enhanced chest computed tomography (CT) scan of patient after pericardial catheter and right sided chest tube insertion. Pneumopericardium, right sided pneumothorax as well as bilateral mild pleural effusion and collapse consolidation are evident. A: Axial view. B: Parasagittal view

After being transferred to the ICU, a third chest x-ray was performed and the diagnosis of pneumopericardium was confirmed. Also, the patient had marked skin crepitus bilaterally on the anterior chest wall. Considering her aggravating condition and the results of cardiology consultation on the pneumopericardium as well as the developing pneumothorax and pneumomediastinum, a pericardial window was opened by a heart surgeon Then after, a chest CT scan was performed to evaluate the result of the intervention (figure 2). 

Laboratory test results are shown in table 1. According to table 1, the patient’s white blood (WBC) cells and platelet counts increased from the beginning of symptoms until the last day. Other results such as prothrombin time (PT) and partial thromboplastin time (PTT) were normal or slightly elevated (non-significant). Also, lactate de-hydrogenase (LDH) was 552 U/l in day 2 which increased to 920 in last day. Despite the surgical intervention, the Macklin phenomenon was not preventable in this patient. Eventually, the patient expressed cardiopulmonary arrest on the third day and unfortunately expired with no identifiable cause.

**Table 1:** The results of laboratory tests

	**Admission**	**Day 2**	**Day 3**	**Day 5**	**Day 7**	**Day 8**
WBC (×10^3^/mm^3^)	6.1	7.0	8.1	10.5	19.7	26.3
Hb (gr/dl)	9.9	10.0	9.2	8.8	8.4	7.9
Plt (×10^3^/mm^3^)	68	111	134	137	207	174
Urea (mg/dl)	18	24	32	42	42	42
Creatinine (mg/dl)	0.3	0.4	0.4	0.4	0.5	0.6
LDH	N/A	522	632	680	786	920

WBC: White blood cells; Hb: Hemoglobin; Plt: Platelet; LDH: Lactate dehydrogenase

## Discussion

Pneumomediastinum, pneumothorax, and pneumopericardium all result from air escaping from the pulmonary system in the mediastinum, pleura, and pericardium, respectively. The mentioned phenomenon could be due to blunt chest trauma or penetrating injuries to the pharynx, hypopharynx, bronchi, or alveoli. Excessive pressure, Valsalva maneuver, and perforation of the digestive system may also cause pneumomediastinum and subcutaneous emphysema (8). Moreover, air can penetrate to the mediastinum or pleura following certain infections as well. This issue has been mentioned in a rare case report of isolated tension pneumopericardium without pneumomediastinum in a patient with malignancy following a fungal infection (9). Pneumopericardium may also occur in other conditions such as cardiopulmonary resuscitation, pericardiocentesis, and esophageal rupture. Although spontaneous pneumothorax is a self-limiting benign condition, is usually associated with extensive workup and treatment (10, 11). 

However, it has no specific diagnostic or treatment method and managing the patients is case-specific. There are a few case reports of pneumomediastinum and pneumopericardium in patients diagnosed with leukemia and mediastinal tumors (12-13). According to Rao et al., a female patient diagnosed with ALL (during induction phase) showed shortness of breath which after the chest x-ray, pneumopericardium was diagnosed. Moreover, due to the presence of a radiopaque mass in the left lung in CT scan, they hypothesized the fungal infection as the possible etiology. Unfortunately, no follow-up for the patient has been performed by the authors (12). In another study by Showkat et al., a male patient was reported with ALL and history of non-productive cough for five days with the crepitus on the anterior chest wall as well as neck and parotid area (bilateral). In the CT scan, pneumorachis and subcutaneous emphysema have been shown. The laboratory results were normal and the blood, urine, and sputum cultures did not show any cultured organisms. 

The patient underwent conservative management for seven days but showed the respiratory arrest and intubated. (13). Herein, despite similarities, the current reported case expressed some differences with the few available reports regarding this issue. In comparison with Rao et al.’s study, our patient was surely afebrile, while fever was one of the early signs that their patient had expressed. Moreover, they did not mention any laboratory results which could question the spontaneous feature of their findings (12). Also, the patient who was reported by Showkat et al. had a 5-day-history of cough and the other symptoms were added gradually not suddenly (compared to our case) (13). Unfortunately, no acceptable reason as an etiology regarding the mentioned phenomenon was found for our case as well as the other mentioned reports. This rare complication should always be in mind when dealing with patients with leukemia and be treated as mentioned before. It is necessary to act very fast and do the required intervention(s) as soon as possible (similar to this case). Although, the lack of a certain etiology and diagnosis, in this case, kept the treatment method uncertain. However, we lost the patient despite all procedures without identifying the cause. Authors hope that other patients could benefit from various treatment options.

The autopsy could be very helpful in identifying the cause but in our case, it was not performed due to lack of parents’ consent. It does not seem that chemotherapy or ALL per se caused pneumothorax or pneumomediastinum followed by pneumopericardium. This association could be due to the complications following chemotherapy or ALL. Nonetheless, each case is different and can teach us a lesson. Our assessments using the available equipment showed that the patient had no problems while the patient had no signs and symptoms regarding this issue before. However, we still do not know whether ALL, chemotherapy, or their complications caused mentioned phenomenon and eventually death in our patient. Taken together, although pneumomediastinum and subcutaneous emphysema are rare in patients with ALL, we strongly suggest clinicians to consider them in these patients presenting respiratory signs/symptoms for faster action in patients with respiratory signs/symptoms.
